# Effects of transjugular intrahepatic portosystemic shunt treatment of patients with liver cirrhosis and portal hypertension: Case series

**DOI:** 10.1097/MD.0000000000026610

**Published:** 2021-07-09

**Authors:** Dan Zheng, Jiao Yu, Hui Li, Hongying Gan, Jun Wang, Ting Jiang, Huanping Ren, Fan Wu

**Affiliations:** Department of Gastroenterology, The Central Hospital of Wuhan, Tongji Medical College, Huazhong University of Science and Technology, Wuhan Hubei, China.

**Keywords:** case study, liver cirrhosis, portal hypertension, portal vein thrombosis, transjugular intrahepatic portosystemic shunt

## Abstract

**Rationale:**

Transjugular intrahepatic portosystemic shunt (TIPS) is well established as an effective treatment tool for portal hypertension. However, the effects of TIPS in patients with liver cirrhosis and portal hypertension have not been adequately verified in clinical trials.

**Patient Concerns:**

To evaluate the effects of TIPS in patients with liver cirrhosis and portal hypertension with or without portal vein thrombosis (PVT).

**Interventions:**

A total of 55 patients with liver cirrhosis and portal hypertension received TIPS treatment from December 2014 to April 2018 were enrolled. Clinical data, including portal pressure, Child-Pugh score, and relevant complications were recorded.

**Outcomes:**

TIPS was successfully performed in 54 patients. The overall technical success rate was 98.19% without serious technical complications. After TIPS treatment, portal pressure was significantly reduced from 38.13 ± 4.00 cmH_2_O to 24.14 ± 3.84 cmH_2_O (*P* < 0.05). In addition, symptoms including gastrointestinal bleeding and ascites were improved after TIPS treatment. During the 6 to 21-month follow up, hepatic encephalopathy in 15 patients (27.8%), shunt dysfunction in 5 patients (9.3%), rebleeding in 12 patients (22.2%) and deterioration of liver function in 2 patients (3.7%) were recorded. Moreover, there were no significant differences in the rates of rebleeding and hepatic encephalopathy between patients with PVT and the non-PVT group, whereas the occurrence rate of TIPS dysfunction was higher in the PVT group, but not statistically significant.

**Lessons:**

TIPS treatment could alleviate the symptoms of liver cirrhosis and portal hypertension in individuals with or without PVT. However, complications during follow-up should be appropriately noted and addressed with corresponding treatments.

## 1 Introduction

Liver cirrhosis has become one of the major causes of morbidity and mortality worldwide.^[1]^ It represents the irreversible final stage of chronic progressive liver disease of various causes, and is characterized by advanced fibrosis, scarring, and the formation of regenerative nodules leading to morphological changes.^[2]^ These changes are associated with a serious increase of intrahepatic resistance to portal blood flow, which leads to elevated portal pressure.^[2]^ Clinically significant portal hypertension is defined as an increase of hepatic venous pressure gradient to >10 mmHg.^[3]^ Portal hypertension plays an important role in the pathogenesis of many complications of cirrhosis, representing the major cause of morbidity and mortality in cirrhotic patients. Therefore, controlling portal hypertension is particularly important for improving patient outcomes.

Transjugular intrahepatic portosystemic shunt (TIPS) creates a connection between the portal and systemic circulation within the liver and has emerged as an effective and noninvasive treatment tool for portal hypertension and its complications.^[4,5]^ During the TIPS procedure, an expandable metal stent is inserted via the jugular vein between the main intrahepatic branch of the portal vein and a hepatic vein, so that the portal flow is partially diverted directly into the systemic circulation to reduce pressure in the portal system.^[6,7]^ It has been reported that TIPS treatment could depress portal vein pressure in 90% cases.^[5,8]^ Therefore, TIPS intervention has been widely applied to recurrent or refractory variceal bleeding, refractory ascites, acute variceal bleed, hepatic hydrothorax and hepatorenal syndrome.^[9]^ Abundant experience and technical improvements of TIPS, such as covered stents, have ameliorated patient outcome, promoting its extensive application.^[10]^ For example, portal vein thrombosis (PVT), which was historically considered a relative contraindication for TIPS, has been considered an indication in recent years. However, conflicting results across previous studies have been reported.

In the present study, the effects and the related complications of TIPS treatment in patients with liver cirrhosis and portal hypertension with and without PVT were assessed. The current results provide suggestions to clinicians and a basis for wide TIPS application in patients with liver cirrhosis and portal hypertension with or without PVT.

## 2 Methods

### 2.1 Patients

This research was approved by the Ethics Committee of Wuhan Central Hospital affiliated to Tongji Medical College, Huazhong University of Science and Technology. Totally 55 patients with liver cirrhosis and portal hypertension received TIPS intervention at Wuhan Central Hospital from December 2014 to April 2018 were included in the present study.

### 2.2 The transjugular intrahepatic portosystemic shunt procedure

Prior to the TIPS procedure, all patients underwent routine laboratory tests, including blood, urine and stool tests, liver and kidney function tests, serum electrolyte assessment and coagulation test. Patients were classified by the Child-Pugh score (grades A,B, and C), which includes ascites, hepatic encephalopathy, total bilirubin, albumin and prothrombin time.^[11]^ All patients underwent abdominal enhanced computed tomography before and after TIPS treatment.

Patients with upper gastrointestinal hemorrhage were treated with proton pump inhibitors, somatostatin, and antimicrobial agents, including third generation cephalosporins or quinolones. Patients with massive bleeding were treated with fluid infusion or blood transfusion to prevent hemorrhagic shock and maintain hemodynamic stability. The TIPS procedure was performed as previously described.^[12,13]^ The hepatic vein was reached using a TIPS set (RUPS-100, Cook, Cook Inc., Bloomington, IL, United States). An 8-mm diameter e-PTFE covered stent (Viatorr; W.L. Gore & Associates, Flagstaff, Arizona, USA) was placed from the hepatic vein to the portal vein under digital subtraction angiography. Portal vein pressure was measured before and immediately after TIPS stent placement.

### 2.3 Follow-up

All patients were followed up by clinical evaluation, serum laboratory tests, and Doppler ultrasound (Phillips iu22 duplex ultrasound console and C5-1 ultrasound probe) before hospital discharge. Patients were administered L-ornithine L-aspartate (LOLA) to prevent hepatic encephalopathy, antimicrobial agents (such as third generation cephalosporins or quinolones) to prevent infection and other medicines such as reduced glutathione to promote liver health. Patients were routinely evaluated by Doppler ultrasound at 4 weeks, 12 weeks, and 24 weeks after the TIPS procedure. Doppler ultrasound was also performed whenever clinical symptoms indicated stent dysfunction.

### 2.4 Statistical analyzes

Statistical analysis was performed with the SPSS 21.0 software. Data were expressed as mean ± standard deviation, or median with interquartile range (IQR) for non-parametric data. Comparisons were performed by *t*-test and chi-square test for normally distributed and non-normally distributed variables, respectively. *P* < .05 was considered statistically significant.

## 3 Results

### 3.1 General information of participants

Patient characteristics are summarized in Table 1. A total of 55 patients were included in the present study, with 35 males and 20 females. The mean patient age was 59.6 ± 10 years (range, 39–78 years). The etiologies of cirrhosis were hepatitis B in 22 patients, hepatitis C in 7, schistosomiasis in 9, alcoholism in 6, autoimmune liver disease in 5, and unknown in 6. Using the Child-Pugh scale, the severity of liver disease was classified as grades A (19 cases), B (32 cases), and C (4 cases). In terms of complications, there were 3 patients with liver cancer, 20 with PVT, 40 with ascites and 2 with a history of hepatic encephalopathy.

**Table 1:** General information of participants.

Etiology of liver disease	Cases
Hepatitis B	22
Hepatitis C	7
Schistosomiasis	9
Alcoholism	6
Autoimmune liver disease	5
Unclassified	6

Child-pugh classification	Cases
Grade A (5–6 points)	19
Grade B (7–9 points)	32
Grade C (10–15 points)	4

With/without PVT	Cases
With PVT	20
Without PVT	35

Portal pressure (mean ± SD)/ cmH_2_O	
Pre-TIPS	38.13 ± 4.00
Post-TIPS	24.14 ± 3.84

### 3.2 Effects and complications of transjugular intrahepatic portosystemic shunt

**Figure 1:**
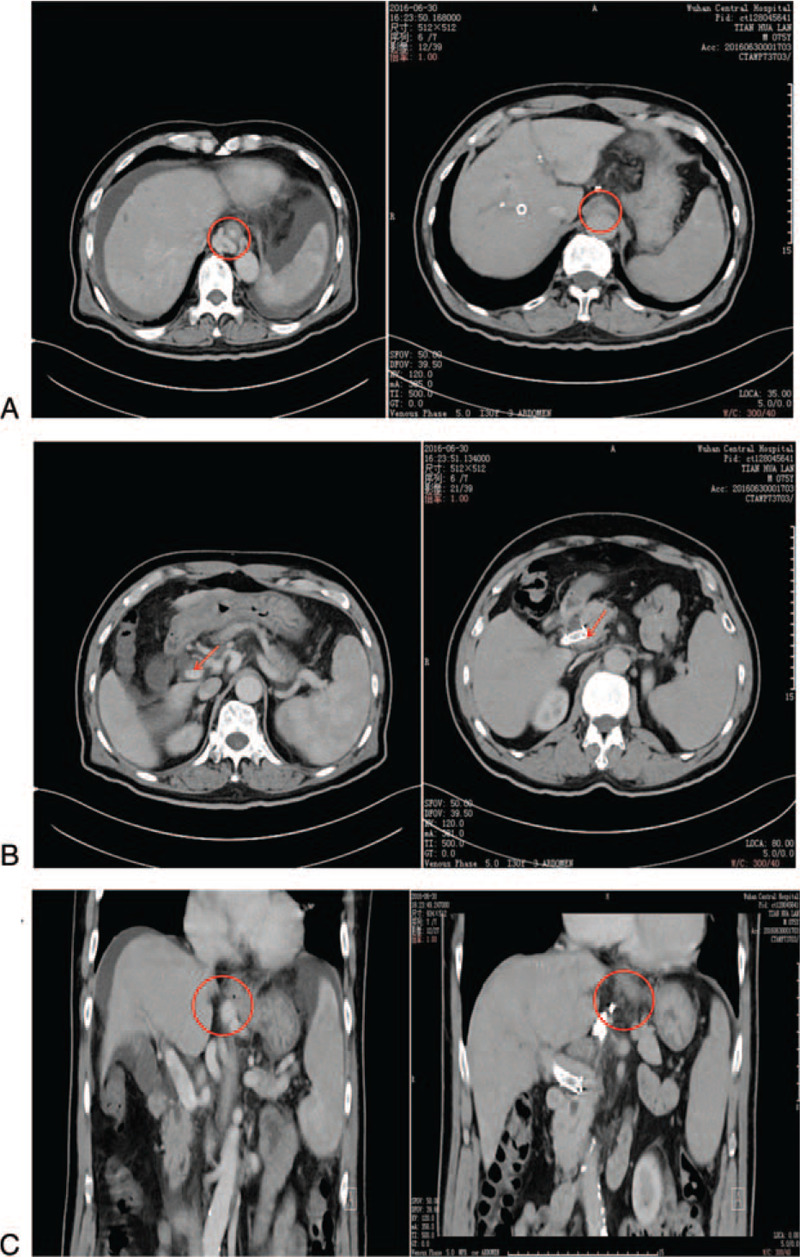
CT scans before and after TIPS treatment. (A). Esophageal varices before (left) and after (right) TIPS treatment. The red circle indicates esophageal varices. (B). Portal vein thrombosis before (left) and after (right) TIPS treatment. The red arrow indicates the portal vein. C. Gastric coronary vein varices before and after TIPS treatment. The red circle indicates the gastric coronary vein. CT = computed tomography.

TIPS was successfully placed in 54 of the 55 participants. The overall technical success rate was 98.19% without serious technical complications. The portal venous pressure was reduced from 38.13 ± 4.00 cmH_2_O to 24.14 ± 3.84 cmH_2_O (*P* < .05) (Table 1). After TIPS treatment, symptoms in patients including gastrointestinal bleeding and ascites were improved. Computed tomography scanning demonstrated that esophageal varices were significantly decreased, and portal vein thrombosis and gastric coronary vein varices disappeared after TIPS treatment (Fig. 1). The patients were followed up for 6 to 21 months after TIPS intervention. The complications of TIPS are summarized in Table 2. We observed hepatic encephalopathy in 15 patients (27.8%), shunt dysfunction in 5 (9.3%), rebleeding in 12 (22.2%), and liver function deterioration in 2 (3.7%).

**Table 2:** Complications of TIPS.

Complications	Number of cases	%
Hepatic encephalopathy	15	27.8
TIPS shunt dysfunction	5	9.3
Acute stent thrombosis	1	1.9
Occlusion	4	7.4
Rebleeding	12	22.2
Shunt dysfunction	5	9.3
Anticoagulant-related gastrointestinal bleeding	3	5.6
Peptic ulcer	2	3.7
Portal venous Thromboembolism	2	3.7
Deterioration of liver function	2	3.7

### 3.3 Transjugular intrahepatic portosystemic shunt complications in patients with and without portal vein thrombosis

To compare the effects of TIPS in patients with and without PVT, we analyzed 3 main TIPS complications. There were no significant differences in rebleeding and hepatic encephalopathy between the PVT and non-PVT groups (*P* > .05). The incidence of shunt dysfunction was higher in patients with PVT, but it was not statistically significant (*P* > .05) (Table 3).

**Table 3:** Compare the main complications of TIPS between patients with and without PVT.

	Patients with PVT (n = 20)	Patients without PVT (n = 34)	***P*** value
Rebleeding	5	7	.74
Hepatic encephalopathy	3	12	.13
TIPS dysfunction	4	1	.06

## 4 Discussion

Liver cirrhosis is associated with higher mortality risk in the presence of portal hypertension.^[14]^ TIPS is a well-established and efficient method for reducing portal hypertension and its complications.^[4,15,16]^ However, the effects of TIPS in patients with liver cirrhosis and portal hypertension have not been adequately verified in clinical trials.

In the present study, the effects of TIPS in these patients were evaluated. We found that TIPS placement was successfully performed in 98.19% participants. There was 1 case in which TIPS placement failed because of the organization of portal vein thrombosis, which greatly increased the difficulty of puncture. After TIPS treatment, the portal venous pressure in the patients was significantly reduced from 38.13 ± 4.00 cmH_2_O to 24.14 ± 3.84 cmH_2_O. Clinical improvement was observed in 54/54 (100%) patients with upper gastrointestinal bleeding (22.2%). Child-Pugh scores were improved in 6 of the 54 patients (11%).

Gastrointestinal bleeding was related to portal hypertension, which is a serious complication of liver cirrhosis. A previous study found that TIPS controls bleeding in >90% of patients and has a rebleeding rate of 12% in individuals with cirrhosis and variceal bleeding.^[15]^ It was reported that in patients with Child-Pugh class C or B cirrhosis showing persistent bleeding by endoscopy, early TIPS treatment is associated with significant reductions in treatment failure and mortality compared with endoscopic and pharmacological therapies.^[15]^ We found that TIPS alleviated bleeding in 100% of patients with a rebleeding rate of 22.2%, which was partially consistent with a previous study.^[15]^ Among the 12 patients with rebleeding, 5 had shunt dysfunction, 3 had anticoagulant-related gastrointestinal bleeding, 2 showed peptic ulcer, and 2 had portal venous thromboembolism. However, their symptoms were improved after the corresponding treatments.

Notably, we observed that 2 major complications might arise following TIPS placement. Hepatic encephalopathy remains a serious postprocedural complication of TIPS intervention. It was reported that the incidence of hepatic encephalopathy following TIPS ranges from 5% to 35%.^[17]^ In this study, 15 of 54 (27.27%) patients developed hepatic encephalopathy after TIPS intervention, which might be mainly due to dietary and bowel habits. After adjusting the dietary and bowel habits, no death occurred in these patients with hepatic encephalopathy. Shunt dysfunction is another common complication of TIPS.^[18]^ Indeed, approximately 50% of patients with bare metal stents require shunt revision during follow-up.^[19]^ However, the use of a new generation of covered expanded polytetrafluoroethylene stents has overcome this problem and significantly improved TIPS patency and clinical efficacy. In this study, 5 cases of shunt dysfunction were recorded, including 4 and 1 due to acute stent stenosis and thrombotic occlusion, respectively.

PVT occurs relatively frequently in patients with liver cirrhosis, and its prevalence increases with disease severity.^[20]^ The presence of PVT has been historically considered a contraindication for TIPS because of a low rate of technical success and a high rate of complications. However, abundant experience and technical improvement have changed this opinion. Several studies have shown that the TIPS procedure could be successfully performed in patients with PVT. In these patients, TIPS could not only restore portal vein patency by direct catheter-directed lysis and removal of thrombus material, but also decompress the portal system by decreasing portal pressure and increasing portal flow velocity.^[16]^ A previous study reported residual thrombus in 77% cases after TIPS placement; however, 76% of cases had complete resolution of thrombus 1 month later.^[21]^ In this study, TIPS was successfully placed in 20 patients with PVT. In terms of complications, there were no differences in rebleeding and hepatic encephalopathy between patients with PVT and the non-PVT group. However, the incidence of TIPS dysfunction was higher in individuals with PVT, but not statistically significant.

There were several limitations in the present study. On the one hand, the participants were limited, which precluded a detailed analysis of all complications associated with the TIPS procedure for a definite conclusion. In addition, it is difficult to obtain the actual mortality risk of TIPS from the presented data. Future large-scale investigations with sufficient long term are required. On the other hand, this was a retrospective study with potential selection bias, and the results might not be comparable with those of controlled studies.

In conclusion, TIPS treatment could improve the symptoms of liver cirrhosis and portal hypertension with or without PVT. However, complications during follow-up should be appropriately noted and addressed with corresponding treatments. These results provide suggestions to clinicians and a basis for wide TIPS application in patients with liver cirrhosis and portal hypertension with or without PVT.

## Acknowledgments

The authors sincerely thank the staff of department of radiology and department of ultrasound of Wuhan Central Hospital who kindly assisted us with follow-up and provided instrumental parameters.

## Author contributions

**Conceptualization:** Fan Wu.

**Data curation:** Hui Li.

**Formal analysis:** Hongying Gan.

**Investigation:** Jun Wang.

**Methodology:** Ting Jiang.

**Resources:** Huanping Ren.

**Writing – original draft:** Dan Zheng.

**Writing – review & editing:** Jiao Yu.
