# Effects of transjugular intrahepatic portosystemic shunt treatment of patients with liver cirrhosis and portal hypertension: Case series: Erratum

**DOI:** 10.1097/MD.0000000000026742

**Published:** 2021-07-23

In the article, “Effects of transjugular intrahepatic portosystemic shunt treatment of patients with liver cirrhosis and portal hypertension: Case series”,^[1]^ which originally appeared in Volume 100, Issue 27 of *Medicine*, the authors have notified the journal of errors in statistical methods. Following a review, the corrections in the article are listed below:

1. In the Abstract, “whereas the occurrence rate of TIPS dysfunction was higher in the PVT group.” is corrected as “whereas the occurrence rate of TIPS dysfunction was higher in the PVT group, but not statistically significant.”
2. In the Statistical Analyses, “Comparisons were performed by the t-test and Mann–Whitney U test for normally distributed and non-normally distributed variables, respectively.” is corrected as “Comparisons were performed by t-test and chi-square test for normally distributed and non-normally distributed variables, respectively.”
3. In the Results, “However, the incidence of shunt dysfunction was significantly higher in patients with PVT (P<.05) (Table 3)” is corrected as “The incidence of shunt dysfunction was higher in patients with PVT, but it was not statistically significant (P>.05) (Table 3)”
4. In the Discussion, “However, the incidence of TIPS dysfunction was significantly higher in individuals with PVT.” is corrected as “However, the incidence of TIPS dysfunction was higher in individuals with PVT, but not statistically significant.”
5. In the Discussion, “We found that TIPS alleviated bleeding in 100% of patients with a rebleeding rate of 18.5%,” is corrected as “We found that TIPS alleviated bleeding in 100% of patients with a rebleeding rate of 22.2%,”
6. In Table 1, the last row reads “Post-TIPS 24.00±5.05”, is now corrected as “Post-TIPS 24.14 ± 3.84”
7. Table 3 is corrected according to the Chi-square test result as follows:

**Table 3:** Compare the main complications of TIPS between patients with and without PVT.

	Patients with	Patients without	
	PVT (n=20)	PVT (n=34)	P value
Rebleeding	5	7	0 .74
Hepatic encephalopathy	3	12	0.13
TIPS dysfunction	4	1	0.06

(Comparisons were performed by chi-square test). The authors apologize for the errors in the published article.
