# Effects of transjugular intrahepatic portosystemic shunt treatment of patients with liver cirrhosis and portal hypertension: Case series

**DOI:** 10.1097/MD.0000000000026679

**Published:** 2021-08-13

In the article, “Effects of transjugular intrahepatic portosystemic shunt treatment of patients with liver cirrhosis and portal hypertension”,^[1]^ a percentage was listed incorrectly in two places. “Clinical improvement was observed in 54/54 (100%) patients with upper gastrointestinal bleeding (18.5%)” should have appeared as “Clinical improvement was observed in 54/54 (100%) patients with upper gastrointestinal bleeding (22.2%).” “We found that TIPS alleviated bleeding in 100% of patients with a rebleeding rate of 18.5%, which was partially consistent with a previous study” should have been listed as “We found that TIPS alleviated bleeding in 100% of patients with a rebleeding rate of 22.2%, which was partially consistent with a previous study.” This does not affect the conclusion.
